# Cutaneous and Ocular Rosacea Associated with Elexacaftor, Tezacaftor and Ivacaftor, A Treatment for Cystic Fibrosis: A Case Report

**DOI:** 10.1016/j.jpedcp.2025.200187

**Published:** 2025-10-10

**Authors:** Audrey Piednoir, Françoise Troussier, Magali Descamps, Laurence Lagarce, Ludovic Martin

**Affiliations:** 1 Department of Dermatology, Angers University Hospital, Angers, France; 2 Department of Pediatrics, Angers University Hospital, Angers, France; 3 Pediatric Ophthalmology, Department of Pediatrics, Angers University Hospital, Angers, France; 4 Department of Pharmocovigilance, Angers University Hospital, Angers, France; 5 Department of Dermatology, Angers University Hospital, Angers, France

**Keywords:** adverse effect, case report, cystic fibrosis, rosacea

## Abstract

A 6-year-old girl with cystic fibrosis developed ocular and cutaneous rosacea after the initiation of elexacaftor-tezacaftor-ivacaftor therapy. Her symptoms improved upon discontinuation and recurred with reintroduction, suggesting a drug-induced reaction. This case suggests a potential, previously unreported association between elexacaftor-tezacaftor-ivacaftor and rosacea, possibly mediated by microbiome alterations and inflammation.

Cystic fibrosis (CF) is an autosomal-recessive genetic disorder that affects approximately 1 in 4500 newborns in France. CF results from sequences changes in the *CFTR* (cystic fibrosis transmembrane conductance regulator) gene, located on chromosome 7. The most common mutation is *F508del*, present in approximately 70% of patients with CF. More than 2000 pathogenic variants of this gene, classified into 6 categories, have been identified that lead to heterogeneous functional consequences. The CFTR protein plays a crucial role in the regulation of ion exchange across epithelial membranes, particularly in the lungs, pancreas, and intestines. The dysfunction of this ion channel impairs water secretion, leading to the thickening of mucus and fluids in various organs, especially in the digestive and respiratory systems. Patients’ quality of life is diminished by complications such as pancreatic insufficiency, liver damage, malnutrition, and recurrent pulmonary infections, which ultimately render the prognosis life-threatening. In addition to these cardinal manifestations, dermatologic conditions may occur in patients with CF. Examples include CF nutrient deficiency dermatitis, aquagenic keratoderma, or cutaneous vasculitis.^1^

Until the 2010s, the management of CF was primarily symptomatic, focusing on airway clearance and the treatment of infections. However, with advances in research and improvements in care, life expectancy has risen to 40-50 years, compared with approximately 5 years in 1960. Among the new treatments, CFTR modulators appear to improve the prognosis of patients with CF.^2^ One such therapy, a combination of elexacaftor, tezacaftor, and ivacaftor (ETI),^3^ was approved for use in Europe in 2020. This triple therapy works by correcting the damaged CFTR protein and enhancing its function. Clinical studies have demonstrated its efficacy in improving lung function and overall quality of life, although a few cutaneous side effects, such as rashes and acne, have been reported.^4^ In this report, we describe a case of both cutaneous and ocular rosacea that occurred in a patient treated with ETI.

## Case Observation

The patient was a 6-year-old girl, born at term with intrauterine growth restriction. She was diagnosed with CF through neonatal screening, revealing heterozygous composite mutations: F508del/c.3196 C > t. She had pancreatic insufficiency and failure to thrive. In June 2023, at the initiation of ETI, she weighed 13.7 kg. Initial sweat test was 131 nmol/kg of sweat. No other treatment was initiated; she was already taking vitamin supplements and pancreatic enzymes. ETI enabled the sweat test to normalize within 1 month. No other concomitant clinical events were reported, and the outcome was favorable in terms of pulmonary function, with a reduction in bronchitis.

At 4 months of treatment, the patient was referred to the Ophthalmology Department because of the development of styes and chalazia, which did not resolve despite the use of topical eye drops. She was also referred to Dermatology at 6 months of treatment for a facial rash. Clinical examination revealed erythematous papules around the nasal area; dry scales in the nasolabial folds, chin, and nasal root; as well as telangiectasias on the cheeks (Figure). A treatment-induced rosacea was suspected. A skin biopsy had not been performed.

**Figure:**
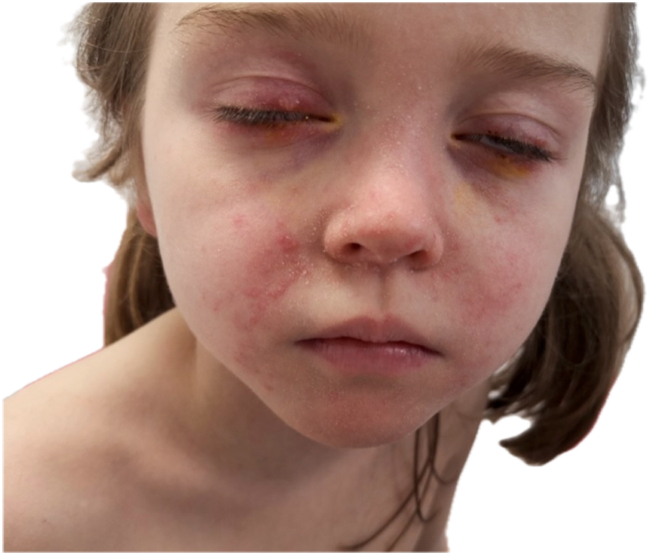
Cutaneous and ocular rosacea. Parental and child consent has been obtained for use of this photograph.

ETI treatment was suspended in January 2024, 6 months after initiation. Topical metronidazole was prescribed, 1 application, then 2 applications per day, and then oral treatment, because of the lack of improvement of the skin lesions. A month and a half after discontinuation, the patient still had faint erythema on her eyelids and dry skin. The granulomatous papules had resolved, leaving behind some postinflammatory pigmentation but no scarring. On ocular examination, more than a month after stopping ETI, the patient showed signs of chronic blepharitis and several chalazia in decline on both upper eyelids. A follow-up visit 2 months after discontinuation revealed complete resolution of the ocular rosacea symptoms.

ETI was reintroduced at a half-dose, 1 tablet of 37.5/25/50 instead of 2, in March 2024. A new rash developed the next day, characterized by papulo-pustules on the cheeks and inflammation of the eyelids. However, the rash showed spontaneous, gradual improvement despite the continuation of therapy. Chalazia also have recurred, requiring long-term treatment with courses of azithromycin, which has proven effective.

## Discussion

The diagnosis of both ocular and cutaneous rosacea attributable to ETI is suspected in this case. Although no previous cases of rosacea have been documented in the context of ETI therapy, its accountability is discussed because of the temporal relationship between the onset of rosacea lesions under treatment, their resolution after discontinuation (dechallenge), and the recurrence of the rash upon reintroduction (rechallenge). This chronological sequence is a classic hallmark of drug-induced conditions and strengthens our argument for a potential association between ETI and rosacea. The international Naranjo algorithm is a standardized scoring system used to assess the likelihood of a causal relationship between a treatment and an adverse drug reaction. In this case, the algorithm yielded a score of 7, indicating a “probable” association.^5^

Demodicosis might be discussed in the differential diagnosis, given its nonspecific clinical presentation can closely mimic various facial dermatoses, including rosacea. Furthermore, the coexistence of *Demodex* infestation in these conditions also can complicate the differential diagnosis.

Cutaneous rosacea has been described in association with several classes of medications, including topical corticosteroids, immunomodulatory agents, and vasoactive drugs such as beta-blockers and calcium channel blockers. The pathophysiologic mechanisms underlying rosacea include immune dysregulation, characterized by increased proinflammatory markers, and vascular changes such as local vasodilation and elevated expression of vascular endothelial growth factor. Genetic predispositions also are recognized, with the condition being more frequently observed in women and individuals of Caucasian descent. However, these mechanisms are not, to date, known with the group of CFTRs. Among the other documented cases of iatrogenic rosacea, there have been instances of spontaneous resolution of symptoms despite the continuation of treatment.^6^ This is consistent with the second episode of rosacea in this patient.

Regarding known side effects of CFTR modulators, acne has been reported multiple times in the literature,^7^ but the underlying mechanisms remain unclear. Hudson et al have proposed that alterations in the skin microbiome, potentially linked to changes in the salt content of sweat, may play a role. Also, chlorine reduction in sweat might lead to an inflammatory skin response.^8^ These 2 hypotheses could intervene in the development of rosacea, a dermatosis in which inflammation plays an important role,^9^ as does colonization by the mite *Demodex folliculorum*.^6^

Further cases are needed to strengthened our hypothesis of a link between rosacea and ETI. Histologic analysis of this type of lesions in patients on ETI could shed light on potential changes in the skin and provide concrete evidence. The identification of granulomatous changes would support the participation of *Demodex*, an important factor in this condition. Considering these findings, we propose to be mindful of facial eruptions with this type of treatment in order to identify similar cases.

## Conclusions

Dermatology may play a role in the management of CF in monitoring and managing the skin-related side effects of therapies like ETI. Early detection of side effects is essential for optimizing patient care and ensuring that the benefits of the treatment outweigh the risks. Therefore, the clinical community should be alerted in front of facial lesions when treating patients with CF who are taking ETI.

## Declaration of Generative AI and AI-Assisted Technologies in the Writing Process

During the preparation of this work the author used ChatGPT (GPT-4) for grammar checking and assistance with rephrasing. After using this service, the author reviewed and edited the content as needed and takes full responsibility for the content of the publication.

## CRediT authorship contribution statement

**Audrey Piednoir:** Writing – review & editing, Writing – original draft, Conceptualization. **Françoise Troussier:** Data curation. **Magali Descamps:** Data curation. **Laurence Lagarce:** Data curation. **Ludovic Martin:** Writing – review & editing, Data curation.

## Declaration of Competing Interest

The authors have no conflicts of interest to disclose.
